# Integrating noncommunicable disease services into primary health care, Botswana

**DOI:** 10.2471/BLT.18.221424

**Published:** 2019-01-08

**Authors:** Neo M Tapela, Gontse Tshisimogo, Bame P Shatera, Virginia Letsatsi, Moagi Gaborone, Tebogo Madidimalo, Martins Ovberedjo, Haruna B Jibril, Billy Tsima, Oathokwa Nkomazana, Scott Dryden-Peterson, Shahin Lockman, Tiny Masupe, Lisa R Hirschhorn, Shenaaz El Halabi

**Affiliations:** a Nuffield Department of Population Health, University of Oxford, Oxford, United Kingdom.; c Division of Global Health Equity, Brigham and Women’s Hospital, Boston, United States of America (USA).; b Ministry of Health and Wellness, Gaborone, Botswana.; c World Health Organization, Gaborone, Botswana.; d Faculty of Medicine, University of Botswana, Gaborone, Botswana.; e Division of Infectious Diseases, Harvard University, Brigham and Women’s Hospital, Boston, USA.; f Department of Immunology and Infectious Diseases, Harvard T. H. Chan School of Public Health, Boston, USA.; g Department of Medical Social Sciences, Northwestern Feinberg School of Medicine, Chicago, USA.; h World Health Organization, Geneva, Switzerland.

## Abstract

Despite the rising burden of noncommunicable diseases, access to quality decentralized noncommunicable disease services remain limited in many low- and middle-income countries. Here we describe the strategies we employed to drive the process from adaptation to national endorsement and implementation of the *2016 Botswana primary healthcare guidelines for adults*. The strategies included detailed multilevel assessment with broad stakeholder inputs and in-depth analysis of local data; leveraging academic partnerships; facilitating development of supporting policy instruments; and embedding noncommunicable disease guidelines within broader primary health-care guidelines in keeping with the health ministry strategic direction. At facility level, strategies included developing a multimethod training programme for health-care providers, leveraging on the experience of provision of human immunodeficiency virus care and engaging health-care implementers early in the process. Through the strategies employed, the country’s first national primary health-care guidelines were endorsed in 2016 and a phased three-year implementation started in August 2017. In addition, provision of primary health-care delivery of noncommunicable disease services was included in the country’s 11th national development plan (2017–2023). During the guideline development process, we learnt that strong interdisciplinary skills in communication, organization, coalition building and systems thinking, and technical grasp of best-practices in low- and middle-income countries were important. Furthermore, misaligned agendas of stakeholders, exaggerated by a siloed approach to guideline development, underestimation of the importance of having policy instruments in place and coordination of the processes initially being led outside the health ministry caused delays. Our experience is relevant to other countries interested in developing and implementing guidelines for evidence-based noncommunicable disease services.

## Résumé

Malgré la charge de morbidité croissante des maladies non transmissibles, l'accès à des services décentralisés de qualité pour lutter contre ces maladies reste limité dans de nombreux pays à revenu faible ou intermédiaire. Dans cet article, nous décrivons les stratégies qui ont été employées pour mener les étapes d'adaptation, de validation et de mise en œuvre à l'échelle nationale des *Lignes directrices 2016 du Botswana sur les soins de santé primaires pour l'adulte*. Ces stratégies ont inclus: une évaluation multiniveau détaillée avec une large implication des parties prenantes et une analyse approfondie des données locales; le recours à des partenariats universitaires; la promotion de l'élaboration d'instruments politiques propices; l'intégration de lignes directrices portant spécifiquement sur les maladies non transmissibles dans les lignes directrices générales sur les soins primaires, en écho à l'orientation stratégique du ministère de la Santé. Au niveau des établissements de santé, les stratégies ont inclus: la création d'un programme de formation multiméthode à destination des prestataires de soins; l’exploitation de l'expérience acquise dans la prise en charge du virus de l'immunodéficience humaine et l’implication des prestataires de soins très tôt dans le processus. Grâce aux stratégies employées, les premières lignes directrices nationales sur les soins de santé primaires ont été validées en 2016, et une étape de mise en œuvre graduelle, sur trois ans, a commencé en août 2017. De plus, la prestation de soins de santé primaires contre les maladies non transmissibles a été incluse dans le 11^e^ plan national de développement du pays (2017-2023). Pendant la phase d'élaboration des lignes directrices, nous avons constaté toute l'importance, dans les pays à revenu faible et intermédiaire, de pouvoir compter sur de solides compétences interdisciplinaires en matière de communication, d'organisation, de création de coalitions et de réflexion systémique et d'obtenir une bonne compréhension technique des meilleures pratiques. Nous avons par ailleurs observé des retards provoqués par des problèmes d'incompatibilité d'agendas entre les différentes parties prenantes, exagérés par des approches cloisonnées lors de la phase d'élaboration des lignes directrices, par la sous-estimation de l'importance d'avoir des outils politiques déjà en place et par des difficultés de coordination des processus initialement pilotés hors du ministère de la Santé. Notre expérience peut être utile pour d'autres pays qui souhaiteraient élaborer et mettre en œuvre des lignes directrices pour des services de soins contre les maladies non transmissibles fondés sur des données probantes.

## Resumen

A pesar de la creciente carga de las enfermedades no transmisibles, el acceso a servicios de calidad descentralizados para estas enfermedades sigue siendo limitado en muchos países de bajos y medianos ingresos. A continuación, describimos las estrategias que empleamos para impulsar el proceso desde la adaptación a la aprobación nacional y la implementación de las directrices de atención primaria de la salud para adultos de Botswana de 2016. Las estrategias incluían una evaluación detallada a varios niveles con amplias aportaciones de las partes interesadas y un análisis a fondo de los datos locales; el aprovechamiento de las asociaciones académicas; la facilidad para elaborar instrumentos normativos de apoyo; la incorporación de directrices sobre las enfermedades no transmisibles en las directrices más amplias sobre la atención primaria de la salud, de conformidad con la dirección estratégica del Ministerio de Salud. A nivel de los centros de salud, las estrategias incluían la elaboración de un programa de capacitación multimétodo para los proveedores de servicios de salud, el aprovechamiento de la experiencia en la prestación de servicios de atención del virus de la inmunodeficiencia humana y la participación de los encargados de la ejecución de los servicios de salud en las primeras etapas del proceso. Gracias a las estrategias empleadas, en 2016 se aprobaron las primeras directrices nacionales de atención primaria de la salud del país y en agosto de 2017 se inició una aplicación por etapas de tres años. Además, la prestación de servicios de atención primaria de la salud para las enfermedades no transmisibles se incluyó en el 11º plan nacional de desarrollo del país (2017-2023). Durante el proceso de desarrollo de las directrices, aprendimos que eran importantes las buenas habilidades interdisciplinarias en comunicación, organización, formación de coaliciones y pensamiento sistémico, así como la comprensión técnica de las mejores prácticas en los países de ingresos bajos y medios. Por otra parte, las agendas desalineadas de las partes interesadas, exageradas por el enfoque aislado del desarrollo de las directrices, la subestimación de la importancia de contar con instrumentos de política y la coordinación de los procesos que inicialmente se llevaban a cabo fuera del ministerio de salud causaron retrasos. Nuestra experiencia es relevante para otros países interesados en desarrollar e implementar directrices para servicios de enfermedades no transmisibles basados en la evidencia.

## ملخص

على الرغم من ارتفاع عبء الأمراض غير المعدية، إلا أن الحصول على الخدمات اللامركزية ذات الجودة العالية للأمراض المعدية ظل محدوداً في العديد من الدول منخفضة الدخل ومتوسطة الدخل. نحن هنا نصف الاستراتيجيات التي استعنا بها لدفع العملية من مرحلة التكيف إلى مرحلة التأييد الوطني وتنفيذ *إرشادات الرعاية الصحية الأولية للبالغين في بوتسوانا لعام 2016 * . وشملت الاستراتيجيات تقييم مفصل متعدد المستويات بمدخلات واسعة من الجهات المعنية وتحليل متعمق للبيانات المحلية؛ والاستفادة من الشراكات الأكاديمية؛ وتسهيل تطوير أدوات دعم السياسات؛ ودمج إرشادات الأمراض غير المعدية في إطار إرشادات الرعاية الصحية الأولية الأوسع نطاقاً مع مواكبة التوجه الاستراتيجي لوزارة الصحة. وعلى مستوى المرافق، شملت الاستراتيجيات وضع برنامج تدريبي متعدد المناهج لمقدمي الرعاية الصحية، والاستفادة من تجربة توفير الرعاية لفيروس نقص المناعة البشرية، وإشراك منفذي الرعاية الصحية في مرحلة مبكرة من العملية. ومن خلال الاستراتيجيات المتبعة، تم اعتماد أول إرشادات للرعاية الصحية الأولية الوطنية في الدولة في عام 2016، وبدأ التنفيذ المرحلي لمدة ثلاث سنوات في أغسطس/آب 2017. وبالإضافة إلى ذلك، فإن تقديم الرعاية الصحية الأولية لخدمات الأمراض غير المعدية قد تم إدراجه في خطة التنمية الوطنية رقم 11 للدولة (2017 إلى 2023). خلال عملية تطوير الإرشادات، أدركنا أهمية المهارات القوية متعددة التخصصات في مجال الاتصالات، والتنظيم، وبناء التحالفات، والتفكير في النظم، والفهم التقني لأفضل الممارسات في الدول منخفضة الدخل ومتوسطة الدخل. وعلاوة على ذلك، فإن الأجندات غير المنسقة للجهات المعنية، والتي يُبالغ فيها نتيجة الأسلوب المنعزل لتطوير الإرشادات، والتقليل من أهمية وجود أدوات للسياسة قيد التنفيذ، وتنسيق العمليات التي تتم بشكل مبدئي خارج وزارة الصحة، قد تسبب ذلك كله في حدوث تأخيرات. تناسب خبرتنا الدول الأخرى المهتمة بتطوير وتنفيذ الإرشادات الخاصة بخدمات الأمراض غير المعدية القائمة على الأدلة.

## 摘要

由于非传染性疾病负担的日益加重，诸多低收入和中等收入国家获得优质、分散式的非传染性疾病服务的途径仍然有限。本文中，我们描述了所采用的策略，该策略推动*《2016 年博茨瓦纳成人初级卫生保健指南》*从适应实际情况到获得国家认可和实施的过程。这些策略包括了纳入广泛利益相关者的意见和对当地数据深入分析基础上的多层次详尽评估；利用学术合作伙伴关系；促进支持性政策工具的制定；将非传染性疾病指南纳入更广泛的初级卫生保健指南，并与卫生部的战略方向保持一致。在医疗机构层面，策略涵盖了为医疗护理提供人员制定的一种多方法培训计划、利用人体免疫缺陷病毒护理提供的经验以及在早期过程中聘请医护人员。通过采用此类策略，该国首个国家初级卫生保健指南于 2016 年获得批准，并于 2017 年 8 月开始分阶段进行三年实施计划。此外，该国第 11 个国家发展计划 (2017-2023) 还涵盖了提供非传染性疾病服务的初级卫生保健服务。在指南制定过程中，我们了解到，在沟通、组织、联盟建设和系统思考，以及对低收入和中等收入国家的最佳实践掌握技术等方面强大的跨学科技能至关重要。此外，利益相关者的议程方向偏离、指南制定的孤立方法过于夸大、制定政策工具的重要性被低估以及最初卫生部以外领导协调流程造成了延误。我们的经验关乎其他有兴趣制定并实施循证非传染性疾病服务指南的国家。

## Резюме

Несмотря на растущее бремя неинфекционных заболеваний, доступ к качественному децентрализованному медицинскому обслуживанию в связи с этими заболеваниями в странах с низким и средним уровнем дохода остается ограниченным. В статье описаны стратегии по содействию данному процессу, начиная с адаптации и заканчивая принятием и внедрением на национальном уровне *рекомендаций по первичному медико-санитарному обслуживанию взрослого населения в Ботсване на 2016 год*. Стратегии включали: подробную многоуровневую оценку с привлечением широкого спектра партнеров и с глубоким анализом местных данных, обеспечение академического сотрудничества, содействие разработке сопутствующих стратегий и правил, внедрение рекомендаций относительно неинфекционных заболеваний в общие рекомендательные документы в сфере первичного медико-санитарного обслуживания с соблюдением основных направлений развития, принятых Министерством здравоохранения страны. На уровне учреждений здравоохранения стратегии включали в себя: разработку многосторонней программы обучения сотрудников системы здравоохранения, эффективное использование опыта, накопленного в ходе выполнения программ по лечению вируса иммунодефицита человека, и привлечение непосредственно занятого оказанием помощи медперсонала на самых ранних этапах. Благодаря этим стратегиям первые национальные рекомендации по первичному медико-санитарному обслуживанию были одобрены в 2016 году, а в августе 2017 года был запущен процесс их поэтапного внедрения в течение трехлетнего периода. Кроме того, оказание первичного медико-санитарного обслуживания применительно к неинфекционным заболеваниям было включено в 11-й национальный план развития (2017–2023 гг.). Процесс разработки рекомендаций продемонстрировал важность вовлечения многопрофильных специалистов в процессы обмена информацией, организации, создания коалиций и системного мышления, а также необходимость практического овладения передовым опытом в странах с низким и средним уровнем дохода. Кроме того, несогласованность интересов партнеров, усугубленная обособленным подходом к разработке рекомендаций, недостаточное понимание важности разработки стратегических планов и координации процессов, которые сначала не подчинялись Министерству здравоохранения, привели к задержкам. Полученный опыт важен для других стран, заинтересованных в разработке и внедрении рекомендаций по медико-санитарному обслуживанию неинфекционных заболеваний, основанному на принципах доказательной медицины.

## Introduction

Noncommunicable diseases cause 41 million deaths each year and accounts for an estimated 71% of all deaths globally.^1^ Of the deaths caused by noncommunicable diseases, 32 million occurred in low- and middle-income countries.^1^ In sub-Saharan Africa in 2015, 34% of all deaths (3.1 million/9.2 million) were due to noncommunicable diseases.^2^ Due to increasing life expectancy, rapid demographic transition and additional risk introduced by human immunodeficiency virus (HIV), the World Health Organization (WHO) estimates that the African Region will experience steep rises in noncommunicable disease incidence and related mortality over the next decade.^3^

However, services to prevent and control noncommunicable diseases in the Region are largely inaccessible or lacking in quality, particularly for poor people and rural residents.^4^^,^^5^There is global consensus that using the primary health-care system, which provides a decentralized and integrated platform of care, is important in addressing noncommunicable diseases.^6^^–^^9^ WHO’s Package of Essential Noncommunicable Disease Interventions (WHO PEN) for primary health care in low-resource settings^10^ provides evidence-based clinical guidelines to improve access and quality of noncommunicable disease services delivered at primary health-care facilities while bolstering the universal health coverage agenda.^2^ Some countries in sub-Saharan Africa have adapted the WHO package to the local context, however few have endorsed them and only two countries, Benin and Togo, have done a national implementation.^2^ However, published experiences from the translation of evidence-based guidelines to routine practice in resource-constrained settings are scarce.^11^ Thus, sharing experiences on implementation of evidence-based guidelines for the delivery of noncommunicable disease services at primary health-care level in such settings is important. Here we describe the strategies employed to drive the process from adaptation to national endorsement of such guidelines and the plan for effective implementation and sustainment of the *2016 Botswana primary healthcare guidelines for adults*.

## Local setting

The burden of noncommunicable diseases in Botswana, a middle-income country in southern African, reflects that of other countries in the Region. In 2014, an estimated 37% (5920/16000) of deaths in the country were due to noncommunicable diseases.^12^ In the same year, a population-based noncommunicable disease risk factors survey conducted among 4074 adults aged 15–69 years showed that an average of 29% (95% confidence interval, CI: 27–32) of participants had hypertension and 18% (95% CI: 16–21) were smoking (Table 1).^15^

**Table 1:** Prevalence of noncommunicable disease risk factors among adults aged 15–69 years, Botswana, 2014

Risk factor	All (*n* = 4074)			Male (*n* = 1321)			Female (*n* = 2753)	
	No.^a^	Weighted % (95% CI)		No.^a^	Weighted % (95% CI)		No.^a^	Weighted % (95% CI)
% of people who currently smoke tobacco	4066	18.3 (15.9–20.7)		1316	31.4 (27.5–35.3)		2750	4.9(3.5–6.2)
% of people with insufficient fruit or vegetable consumption^b^	3651	94.8 (93.4–96.1)		1161	95.8 (93.9–97.6)		2490	93.8 (92.2–95.4)
% of people with insufficient physical activity^c^	3671	20.1 (17.4–22.7)		1182	14.3 (11.3–17.3)		2489	25.9 (22.7–29.2)
% of people who are overweight or obese^d^	3906	30.6 (28.5–32.7)		1299	19.8 (17.0–22.6)		2607	42.3 (39.5–45.0)
% of people with hypertension^e^	4056	29.4 (27.3–31.6)		1314	30.4 (27.2–33.7)		2742	28.4 (25.9–30.8)
% of people with elevated fasting glucose level or currently on treatment for diabetes^f^	3481	4.5 (3.3–5.7)		1115	3.3 (2.2–4.9)		2366	4.8 (3.6–6.1)
% of people who are aged 40–69 years and have a 10-year CVD risk of ≥ 30% or an existing CVD^g^	3468	9.7 (6.9–12.6)		1113	9.3 (5.2–13.5)		2355	10.1 (6.7–13.4)

CI: confidence interval; CVD: cardiovascular disease.

^a^ Denominator of proportion is reported.

^b^ More than five servings of fruit and/or vegetables on average per day.

^c^ Less than 150 minutes of moderate-intensity activity per week.

^d^ Definitions of overweight and obesity are a body mass index of ≥ 25.0 kg/m^2^ and ≥ 30.0 kg/m^2^, respectively.

^e^ People with systolic blood pressure ≥ 140 and/or diastolic blood pressure ≥ 90 mmHg or currently on hypertensive medication.

^f^ Elevated glucose level is defined as a concentration of ≥ 7.0 mmol/L in venous blood.

^g^ A 10-year CVD risk of ≥ 30% is defined according to age, sex, blood pressure, smoking status (smoker defined as current smokers or those who quit smoking less than 1 year before the assessment), total cholesterol and diabetes previously diagnosed or a fasting plasma glucose concentration > 7.0 mmol/L.^13^

Data source: *Botswana STEPS survey report on non-communicable disease risk factors.*^14^

Of the 2 million people living in Botswana, about 95% reside within 8 km of a health facility and basic health services are available for free to all citizens.^16^ The lowest level facilities, the primary clinics and health posts, are each staffed by one or more general nurses, who have at least a two-year nursing diploma following secondary schooling. Botswana’s health-care system shares characteristics with other countries in the Region, including a primary care-based health system structure, shortages in health-care workforce, weak supply chain management and underdeveloped health information systems.^17^^,^^18^

Before 2016, there were no national clinical guidelines for noncommunicable diseases. Adults presenting to primary clinics with a major noncommunicable disease, such as diabetes, hypertension, cardiovascular disease, chronic respiratory disease and cancer, were managed and referred inconsistently, depending on an individual providers’ training or whether the provider used international professional guidelines. In 2013, the health ministry, in collaboration with the University of Botswana, initiated the adaptation of the WHO package for Botswana context,^19^ leading to the endorsement of the country’s first national primary health-care guidelines for adults in November 2016. These guidelines contain standardized algorithms for screening, risk stratification and management of diabetes, hypertension, asthma and screening for, as well as algorithms for broader management of common clinical complaints and preventive care in adults. In addition to evidence-based treatment decision support for health-care providers, the guidelines also emphasize promotion of patient self-management through individual counselling by a nurse and a dietician, as well as group education, defaulter tracing and strengthening coordination of care.

## Approach

**Fig. 1:**
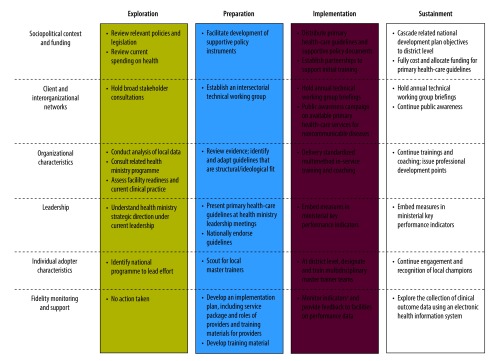
Strategies employed in facilitating endorsement and initial implementation of Botswana’s national primary care guidelines, 2014–2017

^a^ Indicators are based on RE-AIM framework, which assesses five domains: reach; efficacy; adoption; implementation and maintenance^28^

Note: We used the conceptual model for evidence-based practice in public service sectors^20^ to guide the implementation process.

To guide implementation of the guidelines, we selected the conceptual model of evidence-based practice implementation in public service sectors.^20^ We chose this multilevel model, among various dissemination-implementation options,^21^ because of this model’s operational specificity, emphasis on implementation rather than dissemination alone, and relevance to public sector context. The model considers outer, e.g. legislation, policy, funding, and interorganizational networks, and inner, e.g. leadership, organizational culture, readiness for change and individual adopter attitude, including contextual factors that influence the implementation processes. Below we describe, and Fig. 1 outlines, the strategies and processes we undertook during the implementation, using the models’ four phases: exploration, preparation, implementation and sustainment.

### Exploration

To understand the limitations in provision of noncommunicable disease services in the public health-care system, the health ministry’s national noncommunicable disease programme, conducted a multilevel situation analysis. The analysis consisted of a policy and literature review, assessment of available national statistical data, key informant interviews as well as stakeholder inputs from consultative forums. The stakeholders were part of a technical working group that met periodically, and included managers of related national programmes, specialist and general clinicians, academics, hospital administrators and representatives from civil society and development partner organizations.

Before 2016, a national policy or strategy on noncommunicable diseases was lacking. The only national policy instruments related to noncommunicable diseases were the Alcohol Policy, Tobacco Policy, Nutrition Strategy, Essential Health Services Package, and Botswana Public Health Act, and these did not comprehensively address noncommunicable diseases. Published local studies on noncommunicable diseases management at primary health-care level were few, and all were descriptive.^22^^–^^24^ Nonetheless, they indicated gaps in diagnosis, quality of care and control of disease. These findings have been corroborated in analyses conducted following guidelines endorsement (Tapela NM et al., Botswana Health Ministry, unpublished data, 2018; Mosepele M et al., University of Botswana, unpublished data, 2018). For example, analysis of data from the 2014 Botswana STEPS survey on noncommunicable disease risk factors^14^ revealed that 637 of the 1725 participants (weighted percentage: 43%) with elevated blood pressure had not been previously diagnosed with hypertension. Of the 1088 participants with hypertension, 585 (weighted percentage: 53%) had uncontrolled blood pressure (Tapela NM et al., Botswana Health Ministry, unpublished data, 2018). These results are similar to those found in other surveys in the Region,^25^ and indicated that improvement of services for detecting people with hypertension and controlling hypertension was needed. For other chronic noncommunicable diseases such as diabetes and asthma, we hypothesized that the percentages of people with diagnosed disease and the disease under control were also low.

To assess capacity of facilities to deliver essential noncommunicable disease services, we used a self-reported survey derived from WHO Service Availability and Readiness Assessment tool.^26^ We distributed the survey to all 639 primary health-care clinics and 32 district hospitals. Preliminary analysis of the first 142 surveys returned (representing 136 clinics and six hospitals, spanning 10 districts across the country) revealed that essential medicines, basic equipment and relevant laboratory tests were generally available. Furthermore, opportunities for continuing medical education and professional development across professional levels for noncommunicable diseases were lacking. Only six (7%) of the 84 doctors and 27 (2%) of the 1377 nurses surveyed had received any in-service training for noncommunicable diseases management during the previous two years (Government of Botswana, Ministry of Health and Wellness, personal communication, August 2018).

In addition, we visited six primary clinics and two district hospital clinics in two districts to interview key informants and to directly observe consultations for four noncommunicable diseases: cardiovascular diseases; diabetes; chronic respiratory disease and cancers (Tshisimogo G et al., Botswana Health Ministry, unpublished data, 2018). A general physician knowledgeable in primary health-care guidelines observed the consultations and employed a purposive sampling of about 20 consecutive consultations at each facility. Table 2 illustrates findings from observations made for 82 follow-up visits for individuals with hypertension, carried out by 11 health-care providers. Of these consultations, 59 (72%) involved appropriate step up of antihypertensives, that is, initiating new drug or increasing dose for patient reported medication adherence, but still had a blood pressure over 160/100 mmHg. Only 27 (33%) patients received any advice related to healthy diet, physical activity or weight control, and no patients had ever had their body mass index or waist circumference measured at the given facility. These assessments indicated that health-care facilities were generally equipped to provide quality clinical services, but primary health-care providers would need training to effectively implement the guidelines and deliver quality care.

**Table 2:** Observed quality of follow up care for hypertensive patients, Botswana, 2015

Service component	No. of patients (%) *n* = 82
**Patient characteristic**	
With comorbid diabetes	16 (20)
With other noncommunicable disease comorbidities	9 (11)
**Assessment**	
Asked about symptoms	78 (95)
Asked about hospitalization interval	1 (1)
Measured blood pressure	82 (100)
Used correct blood pressure measurement technique	66 (80)
Measured weight	24 (29)
Measured height	0 (0)
Measured waist circumference	0 (0)
Performed foot exam	11 (13)
**Treatment and monitoring**	
Asked about medication adherence	47 (57)
Appropriately increased antihypertensive medication	59 (72)
Ordered appropriate laboratory tests	22 (27)
Scheduled appropriate follow-up	65 (79)
**Education and advise**	
Provided education on disease danger signs	4 (5)
Advised about physical activity	14 (17)
Advised about healthy diet	36 (44)
Advised about alcohol consumption	4 (5)
Advised about tobacco use	2 (2)
Provided any advice on lifestyle modification	27 (33)

### Preparation

To identify options for evidence-based care delivery models in rural or resource-limited settings, we did a web-based literature review by primarily searching PubMed and HINARI, using the search terms “quality improvement” or “guideline implementation”; and “primary care” or “chronic diseases” or “noncommunicable diseases” “healthcare services” or “care”; and “rural” or “resource-limited” or “sub-Saharan Africa”. To select the model that best fitted the context of Botswana’s health system, we considered the alignment with existing national policies and guidelines. We also considered the ongoing transition of the health ministry, which started in 2015, which is a strategic paradigm shift from curative-focused approaches and disease-specific programmes to an emphasis on prevention, early diagnosis and integrated treatment. To gain insights on current practice and potential structural constraints, we held a series of meetings with clinical experts, the Health Ministry Permanent Secretary, manages for HIV, tuberculosis, primary health care and maternal health programmes and clinical services department, and selected clinicians and management staff in district health teams. Based on the findings from the literature review, the key informants deemed the integrated models, such as Wagner’s Chronic Care Model (CCM),^27^ most favourable for the health-care system context. Therefore, the national noncommunicable diseases programme believed that the available WHO package,^10^ underpinned by this Chronic care model, to be the most fitting.

Details of the process of adapting the WHO package to the Botswana context have been previously described.^19^ Briefly, algorithms for screening, risk stratification and/or management of diabetes, hypertension, asthma, breast and cervical cancer were embedded within algorithms for broader management of common clinical complaints in adults (Table 3). The national formulary, which comprises a list of essential medicines covered by the government budget and free to patients, was revised to include relevant medicines from the WHO essential medicine list.

**Table 3:** Outline of the essential noncommunicable disease package included in the *2016 Botswana’s primary health-care guidelines for adults*

Service components^a^	Service task examples	Provider of service
**Patient**		
Education and self-management support	Advise individuals or groups on lifestyle modification, smoking cessation, by employing the five A’s: ask; advise; assess; assist; and arrange	Nurse at primary clinic or dietician^b^
Screening and risk stratification for people older than 40 years	Ask about lifestyle risk factors, including tobacco; harmful alcohol use; diet and physical activity; family history; past medical history; and symptoms related to diabetes, hypertension, heart disease and chronic respiratory disease	Nurse at primary clinic
	Assess age, sex, HIV status, BMI or waist circumference, blood pressure, fasting or random glucose level and total cholesterol level for patients with more than two other risk factors	Nurse at primary clinic
Screening women for cervical and breast cancer	Do pap smear or VIA for females aged 30–49 years and physical breast exam for females aged 40–69 years	Nurse or midwife at primary clinic, VIA performed at district hospital by nurse or midwife
Triage and emergent referral	Assess the criteria for emergent status, such as systolic blood pressure above 200, unstable angina, acute stroke or diabetic ketoacidosis	Nurse at primary clinic, in consultation with nurse^b^ or doctor^b^
Risk-based treatment	For patients with hypertension: initiate antihypertensive if blood pressure is persistently above 140/90; For confirmed diabetes: prescribe metformin and an ACE-inhibitor	Nurse at primary clinic, with initial review by rotating doctor^d^
	Assess 10-year CVD risk^c^	Nurse at primary clinic
	For patients with a CVD risk of 10–20%, suggest lifestyle modifications	Nurse at primary clinic
	For patients with a CVD risk of 20–30%, suggest lifestyle modifications and prescribe statins	Nurse at primary clinic, with initial review by rotating doctor^d^
	For patients with a CVD risk above 30%, suggest lifestyle modifications and prescribe statins and aspirin	Nurse at primary clinic, with initial review by rotating doctor^d^
	Refer patients who have uncontrolled disease despite primary clinic management (e.g. blood pressure > 140/90 despite three antihypertensive medications) to district hospital	Nurse at primary clinic
**Organizational**		
Delivery system design	Trace missed visits and conduct home visits	Nurse at primary clinic, supported by community nurse or social worker^b^
	Provide care coordination support for patients requiring care across facility levels	Community nurse^b^
**Professional**		
Decision support	Train and coach nurses at primary clinics	Master trainer team

ACE: angiotensin-converting-enzyme; BMI: body mass index; CVD: cardiovascular disease; HIV: human immunodeficiency virus; VIA: visual inspection with acetic acid.

^a^ Service components supported by systematic monitoring and evaluation of care, provider training and mentorship, availability of essential medicines and diagnostics.

^b^ Members of the multidisciplinary master trainer team.

^c^ CVD risk is assessed according to age, sex, blood pressure, smoking status (smoker defined as current smokers or those who quit smoking less than 1 year before the assessment), total cholesterol level and diabetes.

^d^ General practitioner seeing patients at district hospital or conducting outreach visits to primary clinics.

The endorsement, effective implementation and impact of the guidelines would depend on a supportive policy environment. Therefore, starting in mid-2015 and concluding in late 2017, development of a multisectoral strategy for the prevention and control of noncommunicable diseases 2017–2022 was accelerated to provide a national roadmap for noncommunicable disease interventions both within and outside the health sector. During the same period, planning for Botswana’s 11th national development plan, for the period 2017–2023, was underway. The health ministry, an actor in this national planning process, identified this timing as opportune. The consultative platforms were leveraged by the health ministry to sensitize stakeholders across sectors, and foster intersectoral action and long-term resource allocation to reduce mortality and morbidity of noncommunicable diseases.

Once we anticipated endorsement of the guidelines, we developed a guidelines implementation plan and training programme for health-care providers with support of a public–private partnership (Box 1). Training materials that were developed were non-proprietary, facilitated by private sector funding and use of readily available software. We used the RE-AIM framework^28^ and additionally WHO HEARTS technical tool^29^ and Partners In Health Guide to Chronic care integration of endemic noncommunicable diseases,^30^ to define a standardized set of performance indicators (Table 4). The national noncommunicable disease programme revised paper-based and basic electronic reporting to include these indicators. Facility staff members reported on these indicators monthly to the district health management teams and national noncommunicable disease programme, using routine district health management reporting practice. On a quarterly basis, the national noncommunicable disease programme compiled and provided feedback of reports data to facilities. A subset of these indicators has been included in key performance targets for the health ministry and in the 11th national development plan.

Box 1 **Curriculum development for multimethod training on primary care-based management of noncommunicable diseases, Botswana**

A multidisciplinary team of clinical experts, many of whom had been involved in the primary health-care guidelines adaptation process, developed the curriculum. Funding and technical support of the curriculum development and training material design, the health ministry established a public–private partnership. The curriculum consists of three modules: (i) risk assessment, diagnosis and treatment; (ii) health education and counselling; and (iii) principles of systems and quality improvement generalizable to chronic conditions, such as longitudinal documentation, missed visit tracing and responding to medicine stock outs.

The curriculum for master trainers included a fourth module on how to be a trainer, encompassing principles in adult learning, mentorship and team-based work. Trainings were intended for maximum 30 participants, with trainer:trainee ratio of 1:10 at most. Training employed several pedagogical methods, including participatory didactic sessions, focus group discussions, practical skills training (such as diabetic foot exam) and role-plays for communication and counselling.

Training of master trainers was five days long and included one clinical nurse (in the first phase of implementation, the nurse was from a comprehensive diabetes clinic), one community nurse or social worker, one medical officer and one dietician from each district. Subsequently, these master trainer teams would lead three-day general trainings in their respective districts (for a minimum of two primary care providers per facility trained in each district) and offer long-term phone-based and site-visit mentorship to health providers at primary clinics.

To evaluate the training, a team from the health ministry’s national noncommunicable disease programme performed surveys before and after training, assessing the participants’ knowledge, skills and confidence in managing conditions. In addition, observation of trainee performance in role plays gave the trainees immediate feedback and if needed, the trainers provided additional practice.

**Table 4:** Key noncommunicable disease performance indicators for Botswana's national primary health-care guidelines implementation

District-level indicator by implementation outcome^a^	Target
**Adoption**	
% of facilities with ≥ 2 providers trained	> 90%
**Maintenance**	
% of facilities with ≥ 2 consecutive monthly reports submitted to district monitoring and evaluation team	> 90%
**Reach**	
% increase in individuals enrolled in care, compared with baseline^b^	> 10%
Coverage of blood pressure screening among residents older than 40 years	> 10%
Coverage of cervical cancer screening among female residents aged 30–49 years	> 10%
Coverage of screening for breast cancer by physical exam, among female residents aged 40–69 years	> 10%
**Implementation**	
% of new visits by patients aged 40 years or older where CVD risk is assessed and documented^c^	> 90%
% of new visits where patients with 10-year CVD risk above 30% is started on statin	> 90%
% all visits where patients with blood pressure above 160/100 antihypertensives are increased	> 90%
**Efficacy of service provision**	
% people with hypertension with most recent blood pressure < 140/90 mmHg (among enrolled patients with a visit during the previous month)	> 60%^d^
Mean change in systolic blood pressure over the past 12 months for people with hypertension	−5mmHg^d^
% of people with diabetes with most recent glucose or HbA1c level < 8 mmol/L and above 6.5 mmol/L (among enrolled diabetics with a visit during the previous month)	> 60%^d^
% patients enrolled in care^b^ with at least one visit in addition to intake visit (retention)	> 90%

BMI: body mass index; CVD: cardiovascular disease: HbA1c: glycated haemoglobin.

^a^ Indicators are based on RE-AIM framework, which assesses five domains: reach; efficacy; adoption; implementation and maintenance,^28^ WHO HEARTS technical tool^29^ and Partners In Health Guide to Chronic care integration of endemic noncommunicable diseases.^30^

^b^ Patients enrolled in care at baseline are individuals who had at least one visit during the 12 months period before guidelines implementation, were not known to have died or relocated and who meet any of the following criteria: known hypertension or diabetes, older than 40 years, or a 10-year CVD risk above 10%. New patients are those with same clinical criteria as above, enrolled in care during the 12 months following guidelines implementation within the given district

^c^ CVD risk assessment deemed completed if the provider had checked and documented: age, sex, blood pressure, blood glucose level, BMI or waist circumference, tobacco use and human immunodeficiency virus status.

^d^ The target consists of two categories: (i) new diagnosis, patients diagnosed within the past 12 months; and (ii) knowing diagnosis, patients diagnosed over 12 months before end of reporting period.

Notes: Targets to be achieved within 12 months of guidelines implementation start. Facilities submit reports monthly including patient-level data, data are then aggregated across districts and nationally reviewed on quarterly and annual basis. Data will be augmented by periodic purposive audits.

### Implementation

The health ministry planned that the implementation should be done in three phases, by scaling up noncommunicable disease services in 8–10 districts during each phase. The first phase began in August 2017 and involved eight districts where an international nongovernmental organization had established multidisciplinary diabetes clinics at district hospitals in 2012. Within each district, the health leadership assigned health-care providers to a district-based multidisciplinary team of master trainers. To obtain the ideal mix of skills in the team, the leadership consulted with district-based health-care providers and the noncommunicable disease programme. Each team consisted of one doctor, one clinical nurse, one dietician and one community nurse or social worker. The team participated in an intensive five-day multimethod training programme (Box 1). Thus far, 32 master trainers covering eight districts have been trained and are currently providing training and case-management coaching for providers at primary health-care facilities throughout their given district. Implementation at an additional nine districts began in May 2018 and implementation in the remaining 10 districts is planned to start in 2019. The aim is achieving national roll-out by August 2020.

### Sustainment

To foster a sustained system change, much was done and planned in advance. For example, inclusion of guidelines indicators both in the ministerial key performance targets and in the national development plan will support high-level policy prioritization and collective programme accountability. To ensure long-term support and institutionalization of guideline-compliant care, we engaged health-care providers and district health managers early on as part of the preparation process. Additionally, training local master trainers in parallel with development of non-proprietary training material will enable future trainings that do not rely upon external resources. To incentivizing participation by nurses, the guidelines training is accredited for nursing clinical professional development points.

## Lessons learnt

By using and strengthening the country’s primary health-care platform, we have accomplished a positive step towards decentralizing quality health-care services for noncommunicable diseases. Botswana is well placed to demonstrate quality and sustained services because of these guidelines and the political support of the national development plan objectives and accessibility of health-care services.

Many of the strategies we employed took into consideration contextual factors (Table 5). For example, emphasizing the potential threat of noncommunicable diseases reversing health gains made by combatting the HIV epidemic facilitated prioritization of noncommunicable diseases during the exploration phase. The health ministry addressed limited expertise in analysing local data and identifying research evidence, a reality in many health ministries in low- and middle-income countries,^31^ by collaborating with academia. This collaboration enabled in-depth analysis of local data and synthesis of published literature. Instead of a more rigorous and resource-intensive assessment of service provision, we distributed self-reported surveys to facilities and visited purposively selected facilities. These surveys were administered by University of Botswana research fellows affiliated with the national noncommunicable disease programme. Analysis of local data clarified local gaps as well as helped engaging policy decision-makers, who were sceptical that international averaged figures reflected local context. More analyses of these data, including further disaggregation by social determinants of health, should be emphasized to better inform policy and practice.

**Table 5:** Key strategies employed in response to contextual factors during adoption and initial implementation of Botswana primary health-care guidelines

Key implementation strategies by implementation phase^a^	Contextual factors
**Exploration**	
Multilevel assessment to understand sociopolitical landscape, funding, current clinical practice and strategic priorities. Used broad stakeholder inputs; review of policies, legislation, programme reports, local data analysis, and operational research	Concerns that noncommunicable diseases might reverse health gains made when combatting HIV.^b^ Existing national noncommunicable disease programme to spearhead effort^b^
Assessed facility capacity and readiness to deliver quality services at primary health-care level. Used purposive sampling and local university trainees to general local data at lower cost	Constrained resources for rigorous facility and provider and/or client assessment
In-depth analysis of local data, leveraged partnerships with academic institutions	Limited research evidence interpretation and analytical expertise within the health ministry; data available from the 2014 noncommunicable disease risk factors survey^b^
**Preparation**	
Selected and adapted guidelines that fit model of care aligned with health ministry structure and strategic direction. Embedded noncommunicable diseases within primary health-care guidelines, aligning with the health ministry strategic direction and emphasizing integrated primary health-care services for individuals with multiple risk factors and morbidities	Key policy instruments did not exist before 2016; the global advocacy for UHC; the health ministry’s primary care-oriented strategic direction^b^
Engaged future on-the-ground adopters early on, starting with guidelines adaptation, to ensure context appropriate guidelines and facilitate ownership and sustainment	Before these guidelines, the experience and focus of health-care providers was predominantly HIV-focused, thus challenging adoption
Set up a broad technical working group and leveraged intersectoral forums to advocate for national prioritization of noncommunicable diseases and enable development of supportive policy instruments, such as a noncommunicable disease strategic plan, national essential medicines list and a national development plan	Tradition of siloed, disease and/or programme-focused approach to guidelines development
Achieved strong and streamlined stakeholder coordination to minimize fatigue and redundancy, through multiple nonlinear related processes^c^	The small pool of local technical experts presenting risk of meeting fatigue
**Implementation**	
Started implementation in districts with some experience in multidisciplinary chronic disease management	Hospital-based multidisciplinary diabetes clinics established in 2012 in eight districts^b^
Coupled standardized in-serve training programme with long-term mentorship to support continued change in practice	Positive and recent experience with HIV training programme, using master trainers^b^
Monitored standardized performance indicators,^d^ which include process measures to signal early on delayed progress and suggest solutions to address delays	No existing routine reporting of noncommunicable diseases care; cumbersome paper-based reporting
Established public–private partnership to provide technical expertise and expediently obtain funding for initial training	Absence of global funding mechanism for noncommunicable diseases; slow government budget allocation processes
**Sustainment**	
Included noncommunicable diseases mortality reduction priority and strategies in the next national development plan. Selected indicators included in health ministry’s key performance indcators	10th National Development Plan ending in 2016^b^
Developed experienced local master trainers and non-proprietary training material to allow for future trainings without need for external resources	Recent and positive experience with national HIV training programme^b^
Going forward, will explore future electronic monitoring of primary health-care indicators, and regular feedback to providers, which will be critical to ensuring continued high-quality surveillance data	Existing patient-level electronic health information primarily for HIV, tuberculosis and child health

HIV: human immunodeficiency virus; UHC: universal health coverage.

^a^ We used a multilevel model that divides the implementation process into four phases: exploration, preparation, implementation and sustainment.^20^

^b^ Enabling contextual factors.

^c^ Nonlinear related processes were noncommunicable disease strategy development, review of essential medicines list, development of primary care guidelines

^d^ We defined the indicators according to the RE-AIM framework.^28^

The preparation phase, leading up to endorsement of the guidelines, was a lengthy, iterative process and subject to many delays. In retrospect, delays were due to a combination of inner and outer contextual factors, including misaligned agendas of stakeholders exaggerated by conventional siloed and disease-specific approach to guidelines, underestimation of the importance of having policy instruments in place and coordination of the processes initially being led outside the health ministry. Development of Botswana’s noncommunicable disease strategy was an enabling and necessary policy step towards guidelines endorsement. The two-year process of developing the noncommunicable disease strategy provided intersectoral stakeholder engagement that was instrumental for the prominent inclusion of mortality reduction of noncommunicable diseases in the national development plan. The process also helped to bring together individuals across the health ministry’s programmes and sectors, who were relevant to adaptation of the guidelines.

During the preparation phase, the national noncommunicable disease programme needed to coordinate diverse stakeholders, consider efficacy of guidelines and other factors in decision-making, such as strategic alignment, equity and the health ministry capacity of additional health services. The programme also needed to handle multiple nonlinear processes, such as development of policy instruments. The health ministry has had an inadequate capacity for health-care stewardship in general,^32^ and this shortcoming was also seen in the guidelines development process. We found that strong interdisciplinary skills in communication, organization, coalition building and systems thinking, as well as a technical grasp of best-practices in low- and middle-income countries, were particularly important. In Botswana, and in many low- and middle-income countries, these skills should be emphasized and developed as a strategy for improving clinical service delivery.

With regards to implementation, strategies employed were informed by published literature on effective guideline implementation and quality improvement.^11^^,^^33^^–^^35^ Limited clinical knowledge and confidence in noncommunicable diseases management by health-care providers have been described in other low- and middle-income countries.^9^ We addressed these issues by developing a multimethod training coupled with a mentorship programme. Phased implementation leveraged the experience of existing district hospitals with multidisciplinary diabetes teams. These teams, while focused on a single disease and based at district hospitals, had experience managing patients with chronic conditions. They were therefore well placed to serve as mentors and receive patients with complex issues, such as multimorbidity or needing special care, referred from primary clinics. Train-the-trainers model mirrored that of Botswana’s successful national HIV training programme.^36^ The potential synergies of applying relevant HIV experience and resources to noncommunicable diseases decentralization have been described,^37^^–^^39^ and incorporating this approach should be suitable in other African countries.

We had to assess and address health-care workforce limitations. While there were some concerns that primary health-care guidelines would introduce additional unbearable workload, facility readiness assessments revealed that most primary clinics generally completed patient consultations by 2 pm. To further facilitate the work of the providers, we also employed task-shifting. The introduction of master trainer positions, which included 50% routine clinical practice and 50% training and mentorship of primary-care clinicians and nurses, required additional sensitization of facility leadership, such as meetings and workload negations. These positions were modelled after the existing tuberculosis and HIV nurse coordinator position and provide an example that facilitated the master trainer positions’ acceptability among health-care providers and administrators.

### Challenges

While political commitment exists, disbursement of funds has been delayed due to complex bureaucratic procedures involved in budget allocation. This delay has resulted in a decreased implementation pace and failure to execute a national communication campaign to raise public awareness on services made available or improved by the primary health-care guidelines. Both epidemiological surveillance and monitoring of health services are necessary to assess the near and long-term impact of these guidelines, however national surveys can be costly and paper-based monitoring unwieldy. Advocacy is ongoing for more resource-efficient surveillance, by including key noncommunicable disease indicators in large better-resourced national surveys, such as the HIV and population surveys, and consolidating related surveys, such as the noncommunicable disease risk factors and tobacco surveys. Collaborative pilot projects are exploring feasible options for monitoring quality of care using electronic patient-level integrated health information platforms.^40^ Finally, evidence-based guidelines need to be reviewed periodically to ensure alignment with evolving evidence. While HIV guidelines have been updated every two years in Botswana, regular review of other guidelines has been less successful, and a review of the primary health-care guidelines would need to be actively promoted.

## Conclusion

By sharing our experience in adapting, endorsing and implementing evidence-based guidelines for noncommunicable diseases, we hope to help other countries planning to implement health services for noncommunicable diseases. We anticipate that lessons learnt will be relevant to stakeholders of national health programmes. The lessons may provide a road map and implementation insights that inform introduction of a WHO package specifically, or of other clinical guidelines that improve services delivered at primary health-care facilities in similar settings.
